# Oxygen saturation levels and retinopathy of prematurity in extremely preterm infants - a case control study

**DOI:** 10.1186/s12887-023-04278-6

**Published:** 2023-09-08

**Authors:** Varnika Aggarwal, Risha Bhatia, Kenneth Tan

**Affiliations:** 1 Department of Paediatrics, Monash University, Melbourne, Australia; 2 Monash Health, Monash Newborn, Melbourne, Australia

**Keywords:** Infants, Newborn; preterm, Retinopathy of prematurity, Oxygen exposure, Oxygen saturation

## Abstract

**Purpose:**

To investigate the association of risk factors, including oxygen exposure, for developing retinopathy of prematurity (ROP) in preterm infants at increased risk of ROP.

**Methods:**

A case-control study was conducted where each infant born at < 28 weeks gestation with ROP was matched with another without ROP over five years (July 2015 – June 2020). Clinical information about the infants was collected from electronic medical records, including method of oxygen delivery, oxygen saturation (SpO_2_), fraction of inspired oxygen (FiO_2_) and mean airway pressure (MAP) measurements. MATLAB was used for a time-averaged analysis. Stata/SE 16.0 was used for statistical analysis.

**Results:**

123 ROP/non-ROP pairs were included in this study. The time-averaged SpO_2_ analysis showed non-ROP group spent more time in hyperoxia than the ROP group (p < 0.001). The non-ROP group had lower respiratory severity scores and analysis when FiO_2_ > 21% showed that were was no difference in SpO_2_ between the two groups when the infants were receiving oxygen support. Conditional logistic regressions showed neonatal surgery significantly increased the risk of ROP (OR = 1.4347, p = 0.010), while the influence of birthweight (odds ratio of 0.9965, p = 0.001) and oxygen exposure (OR = 0.9983, p = 0.012) on ROP outcome was found to be negligible as their odds ratios indicated no influence.

**Conclusions:**

At times when infants were receiving respiratory support (FiO_2_ > 21%) the SpO_2_ data indicated no difference in SpO_2_ between the ROP and non-ROP groups. Analysis of clinical variables found that neonatal surgery increased the odds of developing ROP.

**Supplementary Information:**

The online version contains supplementary material available at 10.1186/s12887-023-04278-6.

## Background

Preterm infants are at risk for various neonatal morbidities. Supplemental oxygen is crucial for the survival of many premature infants [1]. It helps prevent the effects of hypoxaemia but can also contribute to the development of several diseases such as bronchopulmonary dysplasia (BPD) and retinopathy of prematurity (ROP) [2].

ROP is a leading cause of blindness and is becoming increasingly prevalent [3]. ROP is a disease of prematurity, in which development of the vasculature of the retina is not complete at the time of birth and abnormal vascularisation after birth leads to loss of vision. The primary risk factors for ROP are a low gestational age (GA), with very preterm infants most at risk (GA ≤ 31 weeks), and a low birth weight (BW), with infants born at a BW of ≤ 1500 g most at risk [1].

Oxygen supplementation is also a risk factor and plays an important role in the onset of ROP by influencing growth factors such as vascular endothelial growth factor (VEGF) and insulin-like growth factor 1 (IGF-1). Episodes of hypoxia and hyperoxia cause fluctuations in the levels of these factors and induce hypervascularisation of the retina, leading to ROP [4].

Oxygen levels are measured as oxygen saturations (SpO_2_). The Neonatal Oxygenation Prospective Collaboration (NeOProM) trials advise SpO_2_ targets of 91–95% as an optimum range [5, 6]. While the aim of oxygen targeting is to maintain an infant’s SpO_2_ within an optimal range, this is not achieved at times [7].

There has been an increase in the rate of ROP, particularly in developed countries, with more infants requiring ophthalmological interventions [3]. This increase initiated this study investigating whether there is a difference in the SpO_2_ levels of infants with ROP compared to those without, and whether oxygen delivery and other clinical factors influence the risk of ROP in preterm infants.

This study aims to investigate whether there is a difference between the SpO_2_ levels of infants with ROP and infants without ROP for infants born under 28 weeks gestation. We also aim to use additional clinical information for the infants to determine whether there are any confounding factors that could influence ROP outcome.

### Methods

A retrospective case-control study was conducted comparing infants born at < 28 weeks’ gestation with ROP and without ROP cared for at the neonatal intensive care unit (NICU) at Monash Newborn, in Victoria, Australia. Their SpO_2_, fraction of inspired oxygen (FiO_2_) and mean airway pressure (MAP) data for the first two weeks of life were collected and analysed.

The Monash Health Human Research Ethics Committee reviewed and approved this project under the low-negligible risk (LNR) pathway as a quality assurance (QA) or improvement project. Under the LNR and QA pathways for our institution, there was a waiver for need of written consent from the family of these babies.

### Participant selection

Extremely preterm infants (< 28 weeks GA) born from July 2015 to June 2020 and cared for at Monash Newborn were selected for this study. Infants that were more than one week of age when they transferred to the NICU were excluded. Infants that died within the first two weeks of life were also excluded.

### Case-control pairing

For this study, each infant with ROP (case) was paired with an infant without ROP (control).

The information systems, BadgerNet (Clevermed Ltd, Edinburgh UK) and Cerner Millenium Electrical Medical Records, were used to generate a list of patients at the NICU that met inclusion criteria. A case of ROP was defined as ROP of any severity, based on the ophthalmologist assessment. The control group included infants that had not been diagnosed with ROP. Infants with an unidentified ROP status were excluded. 299 infants fulfilled the participant requirements, with 166 ROP infants and 133 non-ROP infants. ROP cases were classified based on guidelines set by The International Classification of Retinopathy of Prematurity [8].

The case and control infants were paired according to GA. This was done by identifying an ROP infant as case and selecting the next non-ROP infant cared for as the control that had the same GA (± 1 week), while ensuring that infants were only paired if they were born less than 12 months apart. This resulted in 123 case-control pairs (Fig. 6), leaving 43 unpaired ROP infants and 10 unpaired non-ROP infants.

### Data collection

For infants born before October 2019, the scanned NICU observation charts were accessed using the hospital’s scanned medical records (SMR) system (Digital Patient Chart, InfoMedix, Melbourne VIC). For infants born after October 2019, the hospital’s electrical medical records (EMR) (Cerner Corporation PTY Limited, North Sydney NSW) were used to view each patient’s neonatal vital signs. The SMR data had been collected manually by NICU staff.

Hourly SpO_2_ measurements were recorded for each patient in a spreadsheet for the first two weeks of data available for each infant. Where available, pre-ductal SpO_2_ values were recorded.

### Data analysis

#### Analysis of SpO_2_ data

Stata/SE 16.0 (StataCorp. 2019. Stata Statistical Software: Release 16. College Station, TX: StataCorp LLC) was used to conduct the statistical analysis. This included use of classical tests such as the t-test and generation of line histograms and use of the Komolgorov-Smirnov (K-S) test for investigating differences in group distributions.

#### Time-averaged SpO_2_ (area under the curve) analysis

**Fig. 1:**
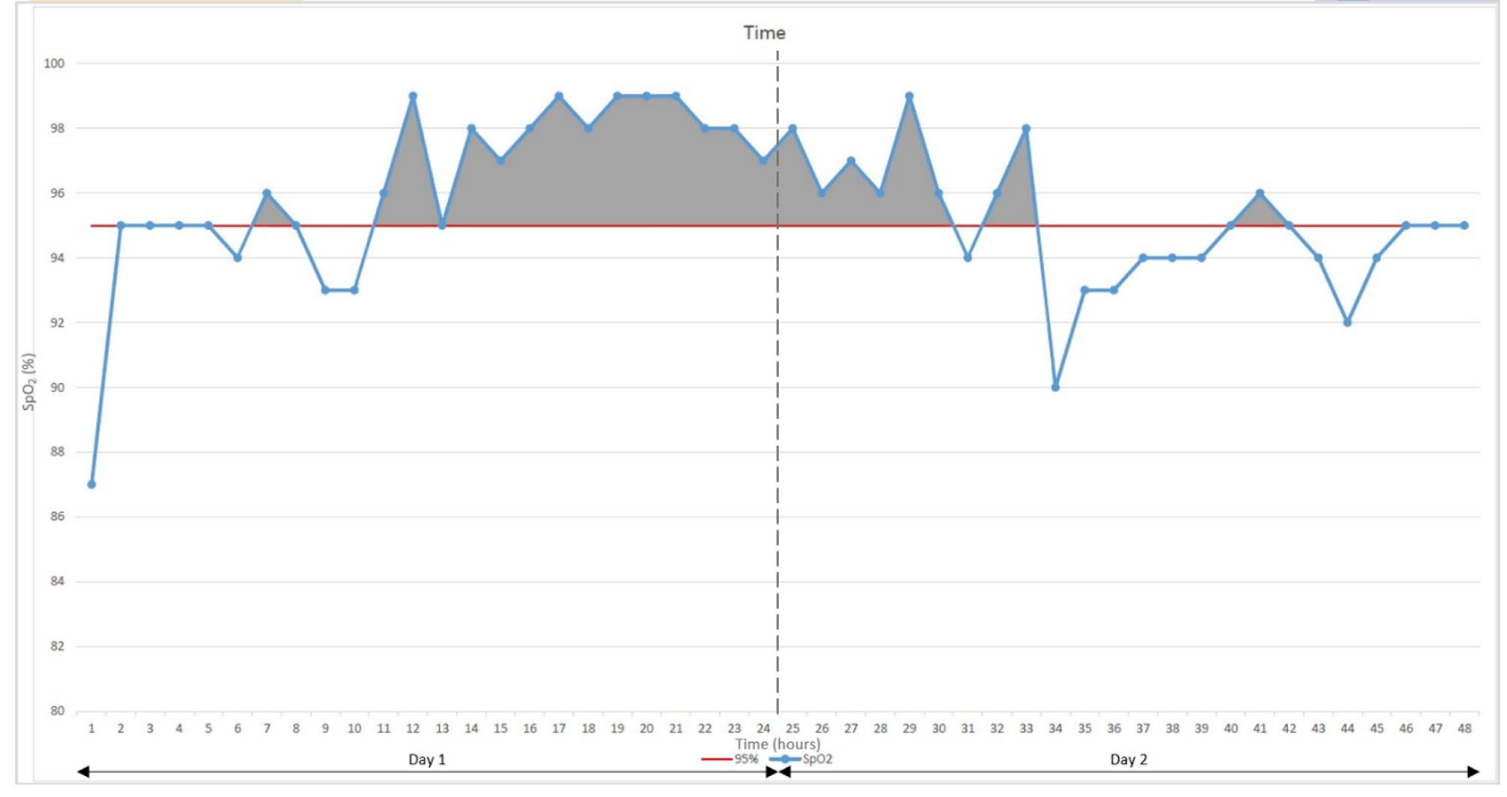
Visual representation of hyperoxic AUC calculated for the time-averaged SpO_2_ analysis. Hyperoxic AUC (grey) shown for one infant over two days as the area between the line graph for hourly SpO_2_ (blue) and the SpO_2_ = 95% line (red)

A time-averaged SpO_2_ analysis was conducted to determine the oxygen exposure of each group. We used the area under the curve (AUC) method, based on a concept used by Okumura et al. [9]. AUC that fell above the SpO_2_ = 95% line for each infant’s SpO_2_ curve was used to find the time spend hyperoxia (Fig. 1).

MATLAB 2020b (Mathworks, Natick MA) was used to find AUC for the SpO_2_ curve of each infant using the trapezoidal numerical integration (*trapz*) function [10].

A two-sampled unpaired t-test was conducted to compare the hyperoxic AUC between the ROP and non-ROP groups for the entire 14 days, only the first week and only the second week. A bar graph was generated (Fig. 4).

Multiple variations of AUC variables were calculated and assessed using a multivariate conditional logistic regression to show if they would affect ROP outcome.

#### FiO_2_ collection and analysis

**Fig. 2:**
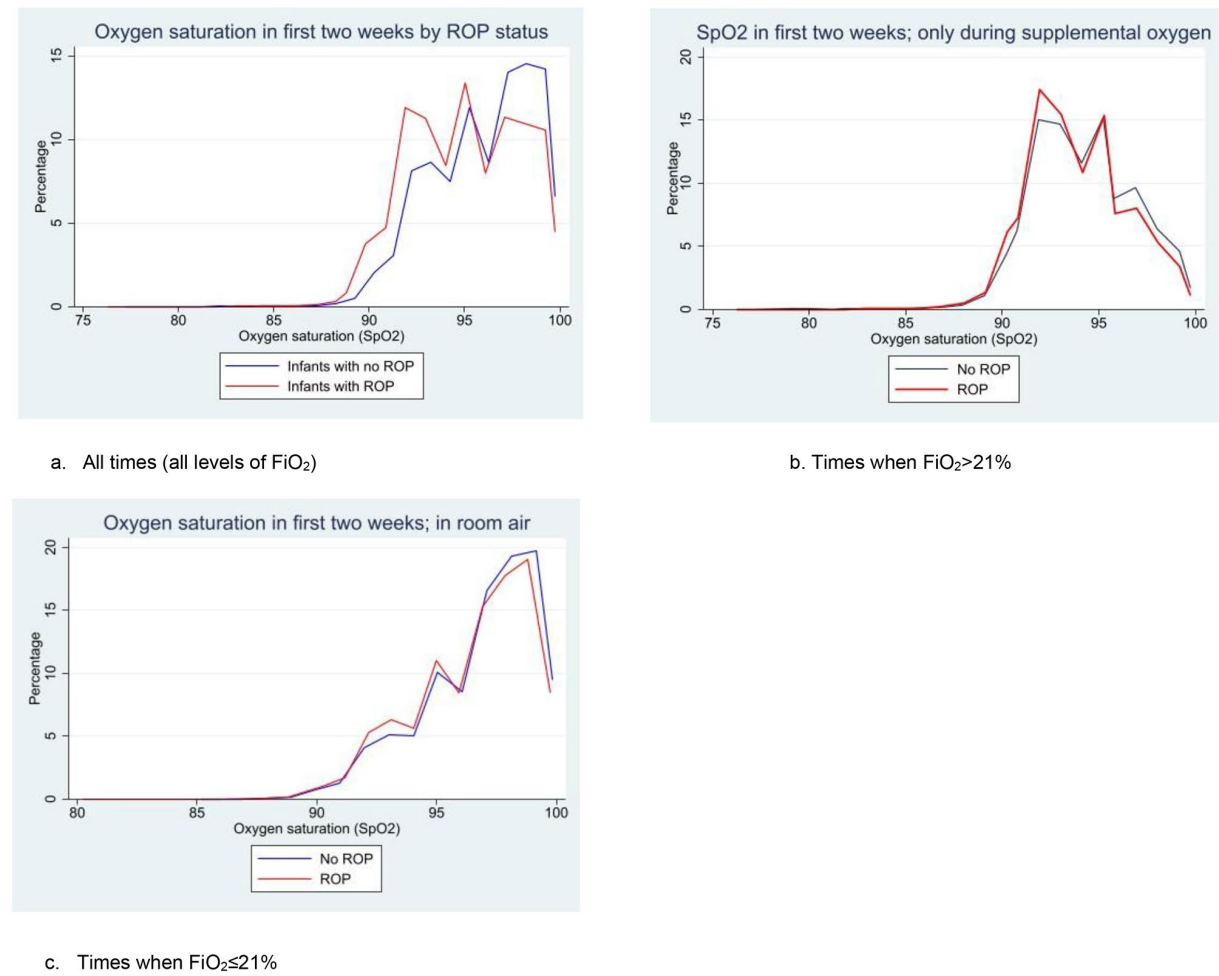
Line histograms comparing the frequency of SpO _2_recordings for infants with and without ROP at (**a**) all times and (**b**) when FiO_2_ > 21% (only during supplemental oxygen), and (**c**) when FiO_2_ < 21% (only during room air). For each respective group, the frequency/percentage is described as a fraction of (**a**) the total number of SpO_2_ observations, (**b**) all SpO_2_ observations when FiO_2_ > 21%, and (**c**) all SpO_2_ observations when FiO_2_ ≤ 21%. Extreme outliers (SpO_2_ < 75%) were excluded

**Fig. 3:**
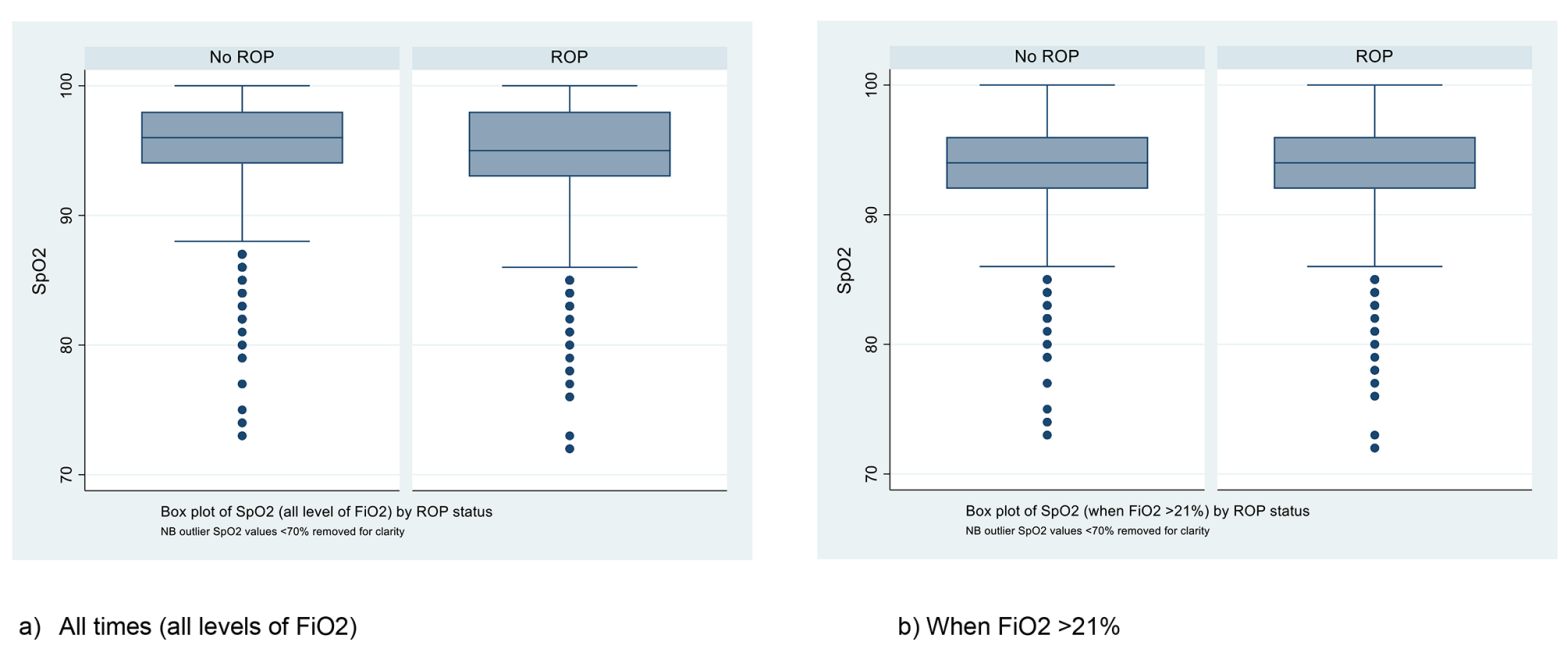
Box plot comparing the distributions of SpO_2_ recordings for the ROP group and non-ROP group at (**a**) all times and (**b**) when FiO_2_ > 21%. Extreme outliers were excluded (SpO_2_ < 70%).

Hourly data on the infants’ fraction of inspired oxygen (FiO_2_) was collected alongside the SpO_2_ data. Using Stata, the times at which FiO_2_ ≤ 21% were removed from analysis. As an FiO_2_ of 21% (or lower) indicates that an infant is in room air, removing such time periods ensure that only the times when infants were receiving supplemental oxygen are analysed. The Komolgrov-Smirnov (K-S) test was conducted again for SpO_2_ levels when FiO_2_ > 21%, and a histogram (Fig. 2b) and boxplot (Fig. 3b) were generated. Separately, the times at which FiO_2_ > 21% were removed from the original analysis and another histogram was generated to show SpO_2_ in room air (Fig. 2c).

Hourly data on the infants’ mean airway pressure (MAP) was collected to determine their respiratory severity scores (RSS) at a given time. RSS was calculated using the following equation: *RSS = (MAP x FiO*_*2*_*)/100*. RSS were calculated using Stata and graphed (Fig. 5). Only RSS for when FiO_2_ > 21% are shown to focus on times at which the infants were receiving oxygen support. A two-sample K-S test and a two-sample Mann-Whitney test were conducted to determine whether there is a significant difference in RSS between the two groups.

#### Mode of oxygenation collection and analysis

Hourly data on the infants’ mode of oxygenation was also collected. The modes of oxygenation were classified as invasive (such as high-frequency ventilation and conventional ventilation) and non-invasive (such as high flow, continuous positive airway pressure and intermittent positive pressure) ventilation. Analysis was conducted using Stata to compare the use of each method of oxygenation between the ROP and non-ROP groups (Fig. 6).

#### Potential confounding variables

Information for the following variables was collected: intrauterine growth restriction (IUGR), antenatal steroids, sex, birthweight, gestational age, exogenous surfactant, nitric oxide, chronic lung disease, postnatal steroids, necrotising enterocolitis, early-onset sepsis, late-onset sepsis, viral infection, surgery and death; to find variables confounding ROP outcomes (Table 1).

**Table 1:** **Demographics and clinical factors in the ROP and non-ROP groups.** This table shows basic descriptive statistics for several clinical variables with respect to the ROP and non-ROP groups, along with the p-value for the difference between the two groups for each variable

Factor	ROP	Non-ROP	P-value
**N**	123	123	
**IUGR, n (%)**	99 (82.5)	98 (80.3)	0.66
**Antenatal steroids, n (%)**	118 (95.9)	122 (99.1)	0.21
**Male sex, n (%)**	57 (46.3)	65 (52.8)	0.31
**Birthweight (g), median (IQR)**	942.0 (830.0, 1058.0)	810.0 (664.0, 927.0)	< 0.001
**Gestational age, median (IQR)**	26.0 (26.0, 27.0)	26.0 (25.0, 27.0)	< 0.001
**Exogenous surfactant, n (%)**	77 (65.8)	101 (84.1)	0.003
**Nitric oxide, n (%)**	4 (3.4)	9 (7.5)	0.17
**Chronic lung disease, n (%)**	76 (61.8)	103 (83.7)	< 0.001
**Postnatal steroids, n (%)**	8 (6.9)	38 (31.7)	< 0.001
**Necrotising enterocolitis, n (%)**	10 (8.3)	18 (15.0)	0.067
**Early-onset sepsis, n (%)**	6 (4.9)	3 (2.5)	0.36
**Late-onset sepsis, n (%)**	28 (22.7)	41 (33.3)	0.17
**Viral infection, n (%)**	7 (5.8)	13 (10.5)	0.5
**Surgery, n (%)**	15 (13.2)	41 (34.1)	0.002
**Death, n (%)**	0 (0.0)	3 (2.4)	0.081

A univariate conditional logistic regression was conducted for these variables with ROP as the dependent variable, excluding gestational age as it had been controlled for via case-control pairing. Variables found to be associated with ROP outcome at a sufficiently significant level (p < 0.25) were selected for further analysis. Total AUC for 14 days was included to represent oxygen exposure as a variable potentially affecting ROP outcome. These were used to conduct a multivariate conditional logistic regression with ROP as the dependent variable (Table S2). The multivariate conditional logistic regression was repeated and non-significant variables (p > 0.05) removed until only birthweight, surgery and total AUC for 14 days remained as the ‘parsimonious’ model. Another conditional logistic regression was conducted for these variables to find their odds ratios (Table 2). Model diagnostics showing measures of fit for conditional logistic regression of ROP were performed to confirm that the statistical model used was good fit for the data.

**Table 2:** **Multivariate conditional logistic regression (odds ratio) results for the parsimonious model (birthweight, surgery and total AUC for 14 days), with ROP as the dependent variable.** The results show neonatal surgery to significantly increase the risk of ROP (OR = 1.4347). The influence of birthweight (OR = 0.9965) and total AUC for 14 days (OR = 0.9983) on ROP outcome was found to be negligible as their odds ratios were very similar to 1, which indicates no influence

Variable	Odds Ratio	95% Confidence Interval	P-value
Birthweight	0.9965	0.9945, 0.9985	0.001
Surgery	1.4347	1.0890, 1.8901	0.010
AUC for 14 days	0.9990	0.9983, 0.9997	0.012

## Results

Out of 299 infants < 28 weeks’ gestation from July 2015 to June 2020, 166 of them had ROP and 133 did not. Pairing based on GA criteria resulted in 123 ROP/non-ROP pairs. The infants had a mean(standard deviation; ±SD) gestational age of 26(± 1) weeks and mean(± SD) birthweight of 867.4(± 212.5) grams.

Basic descriptive statistics were conducted for clinical variables in the context of the ROP outcomes (Table 1). Some descriptive statistics regarding ROP stage and GA can be found in the Supplementary Material (Table S1).

### SpO_2_ data analysis results

A line histogram of the frequency of SpO_2_ measurements for the ROP and non-ROP infants was generated, showing that infants without ROP spent more time in hyperoxia (SpO_2_ of > 95%) than infants with ROP (Fig. 2a). This was supported by results from a two-sample K-S test, which showed that SpO_2_ recordings were significantly higher for the non-ROP group compared to the ROP group (p < 0.001).

However, analysis of SpO_2_ when FiO_2_ > 21% (Fig. 2b) shows no difference between the SpO_2_ measurements of infants with and without ROP. Figure 2c shows that, for both groups, SpO_2_ levels are similarly distributed and are primarily above 95% in room air when FiO_2_ ≤ 21%. The high SpO_2_ levels observed during the times when infants are not oxygenated indicate that they were well enough to have high SpO_2_ levels at these times without the assistance of supplemental oxygen. This indicates that the incidences of hyperoxia shown in Fig. 2a likely occurred at times when the infants were not receiving supplemental oxygen at controlled levels. The p-value for the combined K-S was less than 0.001, indicating a statistically significant difference between SpO_2_ values for the two groups both at all times and when FiO_2_ > 21%.

Descriptive analysis showed that the ROP group had a mean(± SD) SpO_2_ of 95.1%(± 3.1) and the non-ROP group had a mean(± SD) SpO_2_ of 95.9%(± 2.8). The higher standard distribution for the ROP group indicates a marginally wider distribution of SpO_2_ values. Figure 3a also shows a slightly wider distribution of values in the box plot for the ROP group compared to the non-ROP group. However, this is not a notable difference in distribution and should not be used to make implications in practical terms. Considering SpO_2_ levels only during supplemental oxygen shows there is very little variation between the ROP and non-ROP groups (Fig. 3b).

### Time-averaged SpO_2_ analysis results

**Fig. 4:**
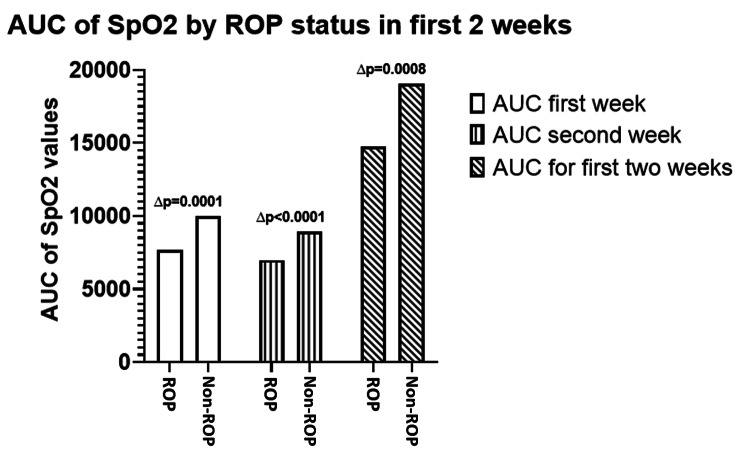
Bar graph showing the mean hyperoxic AUC for the ROP group and the mean hyperoxic AUC for the non-ROP group for the total 14 days, only the first week and only the second week. This bar graph shows that the mean hyperoxic (SpO_2_ > 95%) AUC was higher for the non-ROP group (2) than the ROP group (1) for the total 14 days, as well as only the first week and only the second week. The difference of means (∆p) for each timeframe is indicated

The mean hyperoxic AUC for 14 days, hyperoxic AUC for the first week and hyperoxic AUC for the second week were graphed, showing that the mean hyperoxic AUC was higher in the non-ROP group for all three variables (Fig. 4). The results of a two-sampled unpaired t-test conducted for these three variables show that all the difference of means are significant (p < 0.05) (Fig. 4).

Results from a univariate conditional logistic regression conducted for various AUC variables (e.g., total AUC for 14 days, mean AUC per day, hyperoxic AUC for 14 days, mean hyperoxic AUC per day) show that all AUC variables significantly affect the odds of developing ROP (p < 0.01). The AUC variables were also found to be highly correlated amongst themselves.

### Method of oxygenation analysis results

**Fig. 5:**
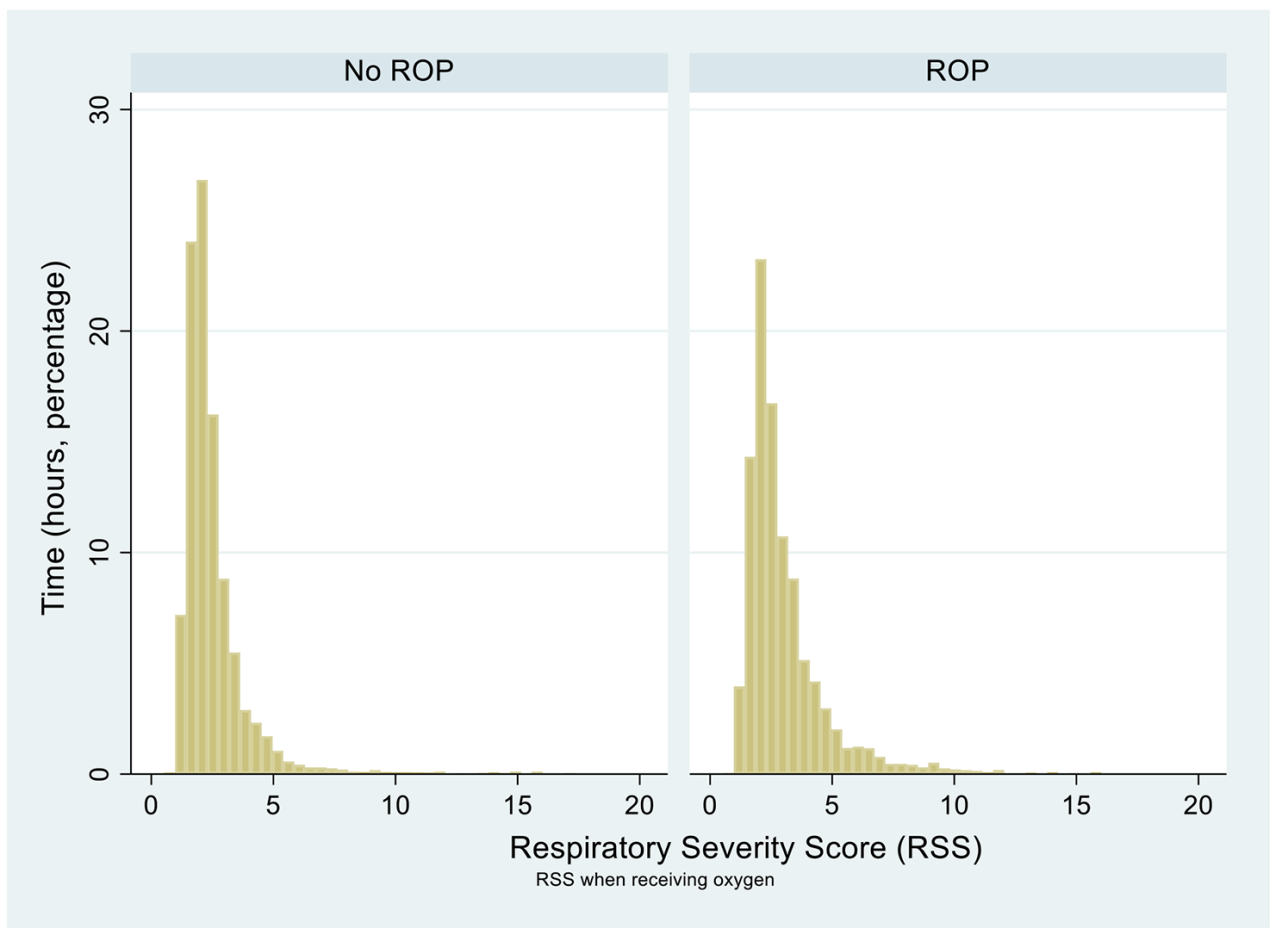
Histogram showing time spent at a certain RSS for the non-ROP and ROP groups for times when FiO2 > 21%. This graph approximates that the non-ROP group spent more time at lower RSS levels than the ROP group

Figure 5 shows that the non-ROP group has a slightly lower RSS than the ROP group, indicating lower levels of respiratory distress. The K-S test and the Mann-Whitney tests show that the non-ROP group has a lower RSS than the ROP group both at all time and at times when FiO_2_ > 21% (p < 0.001).

**Fig. 6:**
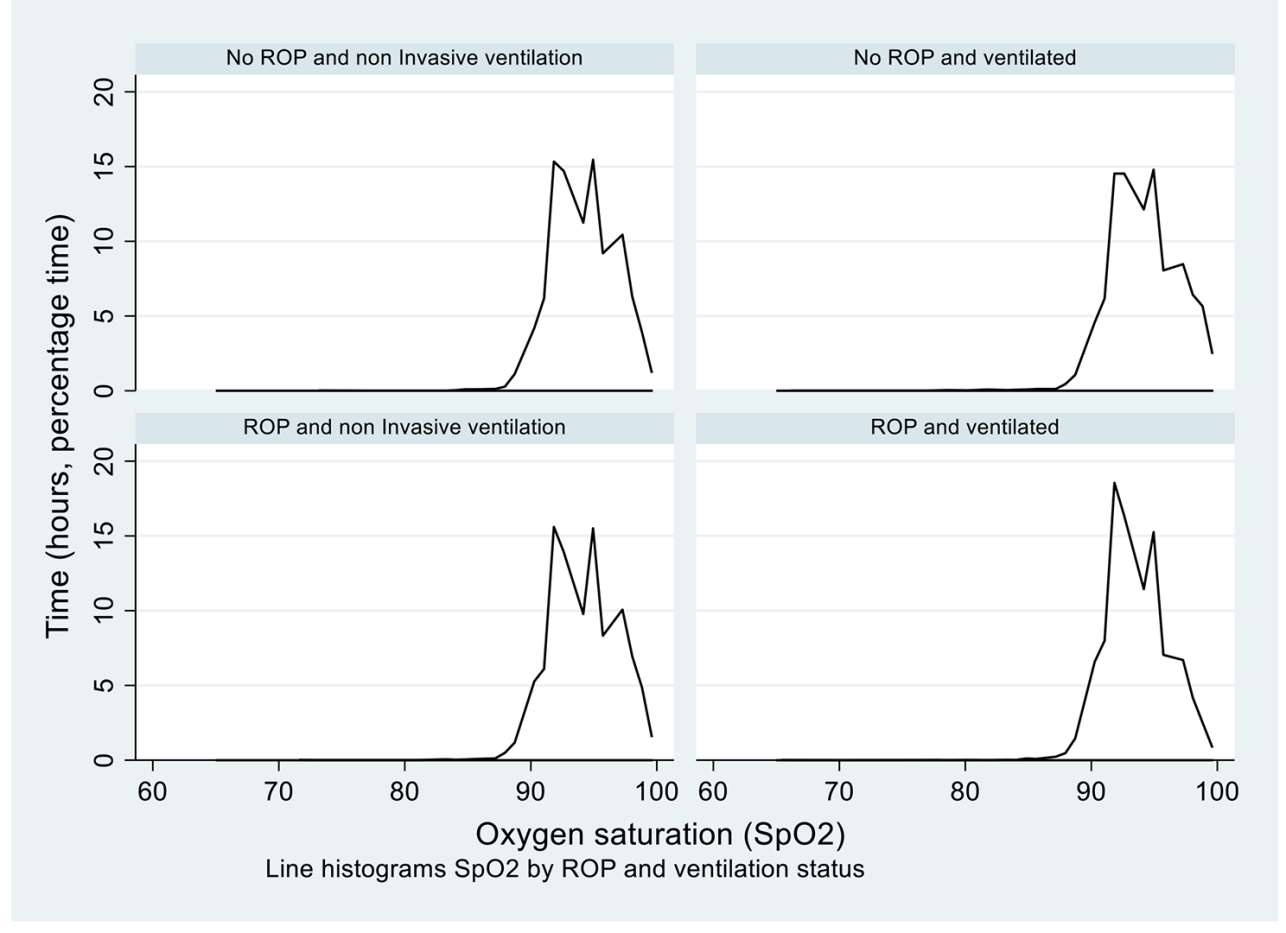
Line histogram showing time spent at SpO_2_ levels for ROP and non-ROP groups, comparing invasive and non-invasive ventilation. The graphs show no notable difference between SpO_2_ levels when comparing invasive and non-invasive ventilation. Extreme outliers were excluded (SpO_2_ < 60%).

Figure 6 shows SpO_2_ levels for the ROP and non-ROP groups according to the time spent using invasive and non-invasive ventilation methods. The graphs are very similar, indicating that use of invasive ventilation compared to non-invasive ventilation did not influence ROP outcome.

### Confounding variables results

Variables found to be significant (p < 0.25) from a univariate conditional logistic regression were used to conduct a multivariate conditional logistic regression, resulting in several variables being non-significant (p > 0.05) (Table S2). After conducting multiple conditional logistic regressions, birthweight, surgery and total AUC for 14 days were found to be the most significant for influencing ROP outcome and were used as for a parsimonious model (Table 2).

The odds ratio and 95% confidence interval for both birthweight and total AUC for 14 days were found to be close to 1. This indicates that, although both their p-values were significant (p < 0.05), birthweight and SpO_2_ AUC did not have any notable influence on ROP outcomes for the infants included in this study. However, as the surgery variable had both a significant p-value (p < 0.05) and an odds ratio and 95% confidence interval that were not close to 1, surgery did influence the odds of preterm infants in this cohort developing ROP. As the odds ratio for surgery was 1.4347, infants who underwent surgery had approximately 43% higher odds of developing ROP.

## Discussion

ROP is a leading cause of blindness and is becoming increasingly prevalent [3]. As high oxygen exposure has previously been associated with an increased risk of ROP, our study focused on whether there is a difference between the SpO_2_ levels of infants with ROP and infants without ROP for infants born under 28 weeks gestation [5, 6].

### SpO_2_ data findings

The main finding the SpO_2_ analysis was that the non-ROP group spend more time in hyperoxia compared to the ROP group. This initial finding was unexpected as the NeOProM trials showed the opposite correlation [5, 6]. However, analysis of SpO_2_ when FiO_2_ > 21% showed that there was no difference between the SpO_2_ measurements of infants with and without ROP while receiving respiratory support. This indicates that the incidences of hyperoxia in the initial analysis likely occurred at times when the infants were not receiving supplemental oxygen at controlled levels. Thus, our findings did not contradict the NeOProM trials as the non-ROP infants in this study had spent more time in hyperoxia because they had more time in room air compared to ROP infants. There were more times when non-ROP infants were well enough to breathe and to have high SpO_2_ levels without the assistance of supplemental oxygen. The non-ROP group was also shown to have lower RSS compared to the ROP group, supporting this explanation. However, while the difference in RSS between the two groups is statistically significant, it is uncertain whether this difference is significant in practice.

### Variables influencing ROP outcome

We found that, for infants included in this study, extremely preterm infants that had undergone surgery had considerably higher odds of developing ROP than infants who had not. Further research, such as a prospective study focusing on the correlation between neonatal surgery and ROP in preterm infants, would allow for a better understanding of how neonatal surgeries may influence ROP onset in preterm infants. RCTs using animal models could be used to determine whether factors related to neonatal surgery may be underlying causes for surgery increasing the risk of ROP.

### Strengths and limitations

The main limitation for this study was the use of one-hourly manual SpO_2_ recordings, as many brief periods of hypoxia or hyperoxia occurring within the hour were likely missed. SpO_2_ data available were manually input by NICU nurses. In addition to the possibility of human error in taking manual recordings, manual data input may lead to underreporting of hypoxic and hyperoxic episodes [11]. Only assessing 2 weeks of data is another limitation as we were unable to account for the infants’ oxygen exposure after these first two weeks, which may have contributed to their ROP outcomes [4].

However, the detailed manual data collection for hourly SpO_2_, FiO_2_, MAP and method of oxygen support data is also a strength of this study as it demonstrates the high level of attention to detail committed to the study. Hourly data spanning two weeks for all 246 infants included in the study amounts to a large pool of data used for the analysis.

The robust analysis is another strength of the study. The time-averaged analysis, especially, is an effective way of measuring and visualising hyperoxia.

The case-control pairing contributed to the small sample size of this study, resulting with 43 ROP infants excluded due to not being paired. These unpaired infants were of a lower GA and higher severity than the paired infants (Table S1), which affects the clinical implications for our results as this highly vulnerable group of infants was excluded from analysis. However, this limitation is difficult to address as case-control pairing was an important part of the study design, allowing us to control for gestational age.

## Conclusion

A retrospective case-control study was conducted comparing oxygen saturation levels between extremely preterm infants with and without ROP. The SpO_2_ data showed that infants without ROP spent more time in hyperoxia than infants with ROP, and that oxygen saturations were more variable for the ROP group, indicating more oxygen desaturations. The ROP and non-ROP groups had similar distributions of SpO_2_ levels at times during which infants were given supplemental oxygen (FiO_2_ > 21%), indicating a lack of strong evidence that supplemental oxygen influenced ROP outcome for infants in this study. It is important to acknowledge that SpO_2_ data was only collected at hourly intervals, likely missing brief periods of hypoxia and hyperoxia. Analysis of additional clinical variables found that neonatal surgery increased the odds of developing ROP in the cohort studied.

Quality improvement processes to audit oxygen delivery are required to identify and address limitations of oxygen targeting in NICUs, and to limit excessive oxygen exposure in preterm infants.

### Electronic supplementary material

Below is the link to the electronic supplementary material.

Supplementary Material 1

Supplementary Material 2

## Data Availability

The datasets generated and/or analysed during the current study are not publicly available due as approval for public availability of datasets was not given by Monash Health’s HREC but are available from the corresponding author on reasonable request, subject to review by the same HREC.
