# Supplementary material for: Oxygen saturation levels and retinopathy of prematurity in extremely preterm infants - a case control study
Source: BMC Pediatr. 2023 Sep 8;23:449. doi: 10.1186/s12887-023-04278-6 (PMC10486104; doi:10.1186/s12887-023-04278-6)
Supplement: Supplementary file 1 — Supplementary Material 1 [file 12887_2023_4278_MOESM1_ESM.docx]

**Table S1. Basic descriptive statistics regarding gestational age (a) and ROP stage (b) for unpaired ROP infants.** There were 43 unpaired ROP infants, with a mean GA of 24 weeks and Stage 3 as the most prevalent ROP stage.

| **(a)** | | **(b)** | |
| --- | --- | --- | --- |
| **Gestational age (weeks)** | **n (%)** | **ROP Stage** | **n (%)** |
| 23 | 3 (6.97) | 1 | 7 (16.28) |
| 24 | 29 (67.44) | 2 | 16 (37.21) |
| 25 | 4 (9.31) | 3 | 19 (44.19) |
| 26 | 2 (4.65) | 4 | 1 (2.32) |
| 27 | 5 (11.63) | 5 | 0 (0.0) |
