# Supplementary material for: Oxygen saturation levels and retinopathy of prematurity in extremely preterm infants - a case control study
Source: BMC Pediatr. 2023 Sep 8;23:449. doi: 10.1186/s12887-023-04278-6 (PMC10486104; doi:10.1186/s12887-023-04278-6)
Supplement: Supplementary file 2 — Supplementary Material 2 [file 12887_2023_4278_MOESM2_ESM.docx]

**Table S2. Multivariate conditional logistic regression (odds ratio) results for nine potential confounding variables and total AUC for 14 days, with ROP as the dependent variable.** Total AUC for 14 days was included to determine the influence of oxygen exposure on ROP outcome.

| **Variable** | **Odds Ratio** | **95% Confidence Interval** | **P-value** |
| --- | --- | --- | --- |
| Birthweight | 0.9972 | 0.9951, 0.9993 | 0.011 |
| Exogenous surfactant | 1.3899 | 0.5755, 3.3567 | 0.464 |
| Nitric oxide | 1.6541 | 0.2501, 10.937 | 0.602 |
| Chronic lung disease | 1.3107 | 0.5308, 3.2363 | 0.557 |
| Postnatal steroids | 2.5844 | 0.7339, 9.1007 | 0.139 |
| Late-onset sepsis | 1.9182 | 0.8064, 4.5629 | 0.141 |
| Viral infection (one) | 7.8416 | 1.0935, 56.231 | 0.040 |
| Surgery | 1.2204 | 0.8662, 1.7194 | 0.255 |
| Parenteral hours | 0.9999 | 0.9992, 1.0006 | 0.941 |
| Total AUC for 14 days | 0.9991 | 0.9982, 1.0000 | 0.066 |
